# Case report: Olaparib as an experimental therapy in a BRCA2-mutated patient with metastatic ovarian adenocarcinoma that originated from liver cancer

**DOI:** 10.3389/fonc.2022.1010158

**Published:** 2022-12-12

**Authors:** Caixia Li, Wenlei Ye, Wenni Zhou, Zhikang Ye, Weihong Yang, Zhongping Cheng

**Affiliations:** Department of Gynecology and Obstetrics, Tenth People’s Hospital Affiliated to Tongji University, Shanghai, China

**Keywords:** secondary ovarian tumor, liver cancer, olaparib, metastasis, case report

## Abstract

Secondary ovarian tumor [secondary tumor of the ovary (STO)] is not a frequent disease. To date, there is still a lack of standard treatment for STO due to the relative heterogeneity. Liver cancer metastasis to the ovary is extremely rare, with only 17 living cases having been reported so far, making it impossible to launch large-scale prospective studies and formulate the standard intervention for patients. We herein report a rare case of STO with liver primary cancer metastasis to the ovary and omentum in a 66-year-old woman. The patient underwent debulking surgery with the removal of the uterus, bilateral fallopian tubes, bilateral ovaries, appendix, and a large part of the omentum majus. Next-generation sequencing was conducted after the operation, identifying BRCA2 mutation. Because strongly refusing chemotherapy, she received olaparib as an experimental therapy. After the administration of surgery and olaparib, the serum value of cancer antigen 125 (CA125) and alpha fetoprotein (AFP) decreased dramatically and basically remained within the normal range. So far, she has achieved nearly 2-year survival and lives a relatively normal life with good quality.

## Background

The ovary is a common site of metastatic tumor, which makes secondary ovarian tumors [secondary tumors of the ovary (STOs)] account for 10%–25% of all ovarian malignancies (1). The common primary tumors that give rise to ovarian metastases include breast, colorectal, endometrial, stomach, and appendix cancer (1). Liver cancer is one of the exceedingly rare malignancies that metastasize to the ovary, with only 17 cases reported in the literature, which was first reported in 1983 (2). The prognosis of patients with ovarian metastasis is generally poor, with a 5-year survival rate of 18.5%. Although it varies among various primary tumors (1), the reported median survival after the diagnosis of extrahepatic metastasis is merely 8.1 months (3). To date, there is no standard management for STOs, although it is believed that they should be treated according to their histological types and stages on the occasion that the primary tumor is verified (4). Some data suggest that cytoreductive surgery following adjuvant chemotherapy may provide a survival benefit for select subgroups of patients with STOs (1).

In recent years, the molecularly targeted drugs have made vigorous progress in tumor precision medicine treatment. Poly adenosine diphosphate-ribose polymerase inhibitors (PARPi) target the NAD+ at the catalytic site of PARP1 and PARP2 to inhibit the function of PARP, which is necessary for the repair of DNA damage (5). Olaparib is an oral PARPi, which can cause synthetic lethality in tumors with homologous recombination repair deficiencies (5). It has been approved as a maintenance therapy for patients with platinum-sensitive recurrent ovarian cancer or *BRCA*-mutated cancer (6). At present, considerable clinical trials are ongoing to evaluate the effectiveness of PARPi in various solid tumors such as gastric cancer, prostate cancer, head and neck cancer, and liver cancer, with some obtaining exciting preliminary data and treatment response.

Herein, we report the case of a 66-year-old woman with rare ovarian and omentum metastases that originated from liver cancer. She was suspicious of STO more than 6 years after the primary surgery for liver cancer in 2013. Then, the patient underwent pelvic debulking surgery in our department in 21 August 2020. The diagnosis of ovarian adenocarcinoma that metastasized from liver cancer was confirmed, considering the medical history and the reports of pathology and immunohistochemistry. The synchronous genetic testing showed that she was associated with BRCA2 mutation. On account of refusing postoperative platinum chemotherapy, she was recommended to have targeted medicine. Olaparib was used as an experimental therapy following cytoreductive surgery. The patient has acquired nearly 2-year survival and remains in good condition. Our therapy experience on this case will shed light on the treatment of secondary ovarian cancer arising from liver cancer or others.

## Case presentation

The patient was a 66-year-old postmenopausal woman. She had pregnancy twice: one time, natural production; another, induced abortion. She had no bad personal habits and customs. The test results for hepatitis B and C were negative. There was no family history of malignancy. She had suffered from primary hepatocellular carcinoma (HCC) and underwent partial resection of the left liver lobe plus the resection of the metastatic lesion in the abdominal cavity without receiving chemotherapy and radiotherapy in June 2013 and has been receiving traditional Chinese medicine since then. Two years after the initial liver cancer, metastases to the lymph nodes were found in the upper abdominal region, and she received 25 courses of radiotherapy.

**Figure 1:**
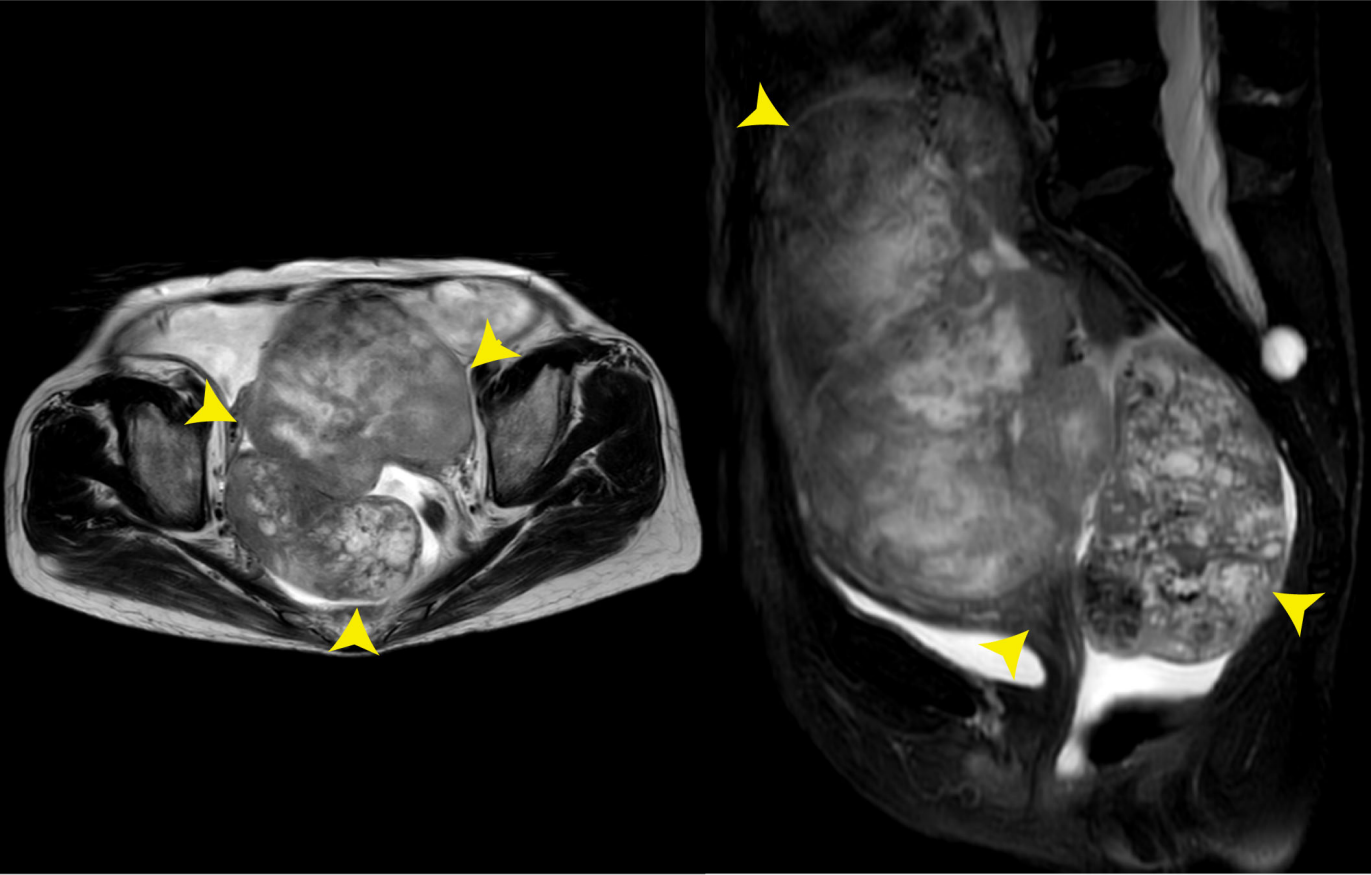
Pelvic MRI on 18 August 2020. The images showed an expansive heterogeneous malignant tumor in the lower abdominal and pelvic cavity (yellow arrows).

In February 2020, she was admitted to a local hospital due to a 6-month history of lower abdominal discomfort and distension, increasing abdominal circumference, but weight loss (5 kg in 1 week). The transabdominal ultrasound (TAS) showed huge solid masses in the pelvic cavity with ascites. Later, the positron emission tomography/computed tomography (PET/CT) scan performed in another hospital showed images of bilateral adnexal masses, which suggest malignant ovarian tumors. However, the probability of metastatic tumor could not be excluded. For further treatment, the patient was referred to our hospital on 18 August 2020. Physical examination revealed large, palpable, fixed, solid, and irregular masses with slight tenderness in the region of bilateral adnexa, which was about 12 cm × 10 cm × 10 cm in the left and 8 cm × 6 cm × 6 cm in the right. Superficial lymph nodes were not enlarged. Shifting dullness was positive. The serum cancer antigen 125 (CA125) level was 301.00 U/ml (the normal value is <35 U/ml), with a dramatic high level of alpha fetoprotein (AFP), which was more than 1,210.00 ng/ml (the normal value is <7 ng/ml). The serum carcinoembryonic antigen (CEA) and CA19-9 values were normal. A dynamic magnetic resonance imaging (MRI) scan of the pelvic cavity was performed and revealed an expansive heterogeneous malignant tumor measuring 19.3 cm × 11.1 cm × 10.9 cm in the lower abdominal and pelvic cavity with a large quantity of ascitic fluid, suggesting the possibility of ovarian malignancy (**Figure 1**). A dynamic CT scan of the upper abdomen showed a 2.6 cm × 2.8 cm × 2.5 cm irregular nodule over the head of the pancreas with the possibility of metastasis accompanied by plenty of ascites and medium quantity of pleural effusion in the left chest. No tumor recurrence in the liver was detected, and no new tumors were found in the kidneys, urinary bladder, or lymph nodes located in the pelvic retroperitoneum. Electronic colonoscopy did not show any tumors in the rectum or colon.

**Figure 2:**
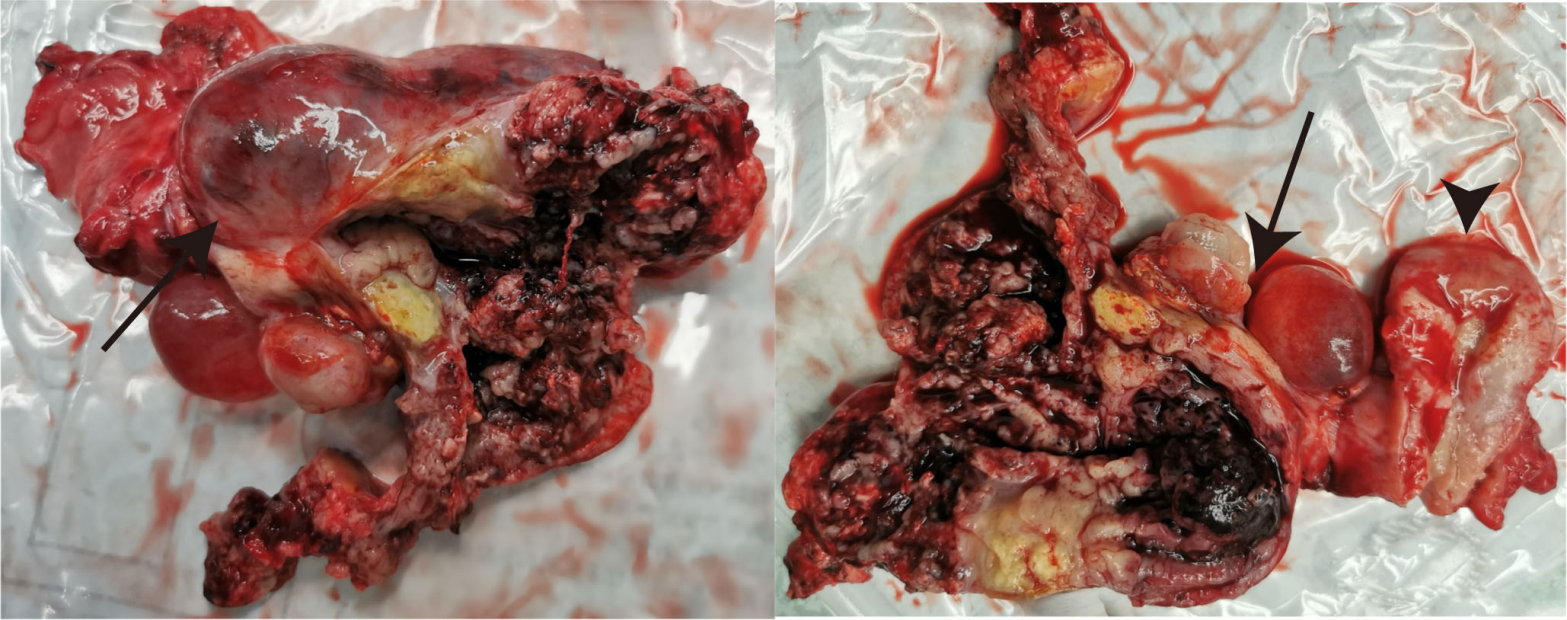
The gross view of the resected tumor originating from the bilateral ovaries (arrows) and uterus (arrowheads).

After the comprehensive assessment including the state of disease and patient’s desire, the decision for tumor excision was made and the surgery was performed on 21 August 2020. There were cystic-solid tumors with uneven surfaces that originated from the bilateral ovaries with left and right ovaries measuring about 12 and 8 cm in diameter, respectively (**Figure 2**). The surface of the diaphragm was smooth, and no visible lesions were found in the urinary bladder, intestine, gallbladder, or liver. The deep red bloody ascites was about 3,000 ml. The intraoperative frozen section histology of the right adnexa revealed ovarian malignancy. Therefore, the patient underwent total abdominal hysterectomy with bilateral salpingo-oophorectomy and appendectomy, and a large part of the omentum majus was also removed.

The results of immunohistochemistry experiments of tumor tissues showed that ER-, PR-, PAX-8-, SALL-4-, WT-1-, CK-pan+, CK8/18+, Villin+, Hepa+, GPC-3+, AFP, and ARG-1 were partially positive. The Ki-67 of the tumor was about 40%. Based on the patient’s medical history and the histopathology report, a final pathologic diagnosis of bilateral metastatic ovarian adenocarcinoma that originated from HCC with omentum majus metastasis was made. About 2 weeks after the surgery, the level of CA125 and AFP decreased to 36.00 U/ml and 821.00 ng/ml, respectively.

After the operation, she received genetic counseling with gave informed consent for testing. The sample was prepared for next-generation sequencing on 25 August 2020. Mutations in exon 11 of *BRCA2* and exon 17 of *RAD50* as well as exon 4 of *PALB2* were identified. The patient refused to receive chemotherapy due to its side effects. Given that the specific mutation was in *BRCA2* and *RAD50*, the PARPi olaparib was used, which began at 300 mg twice daily on 29 September 2020. Unfortunately, she did not tolerate the side effects disturbing her normal life. She felt asthenia after the administration of olaparib with a white blood cell decreasing count of 2.47 × 10^9^/L on 9 November 2020. As a result, olaparib was withdrawn after receiving it for 2.5 months. The patient was on regular outpatient follow-up and is now in good condition. Informed consent for publication of data and images was obtained from the patient. This study was approved by the institutional review board of the Tenth People’s Hospital Affiliated to Tong Ji University.

## Outcome and follow-up

**Figure 3:**
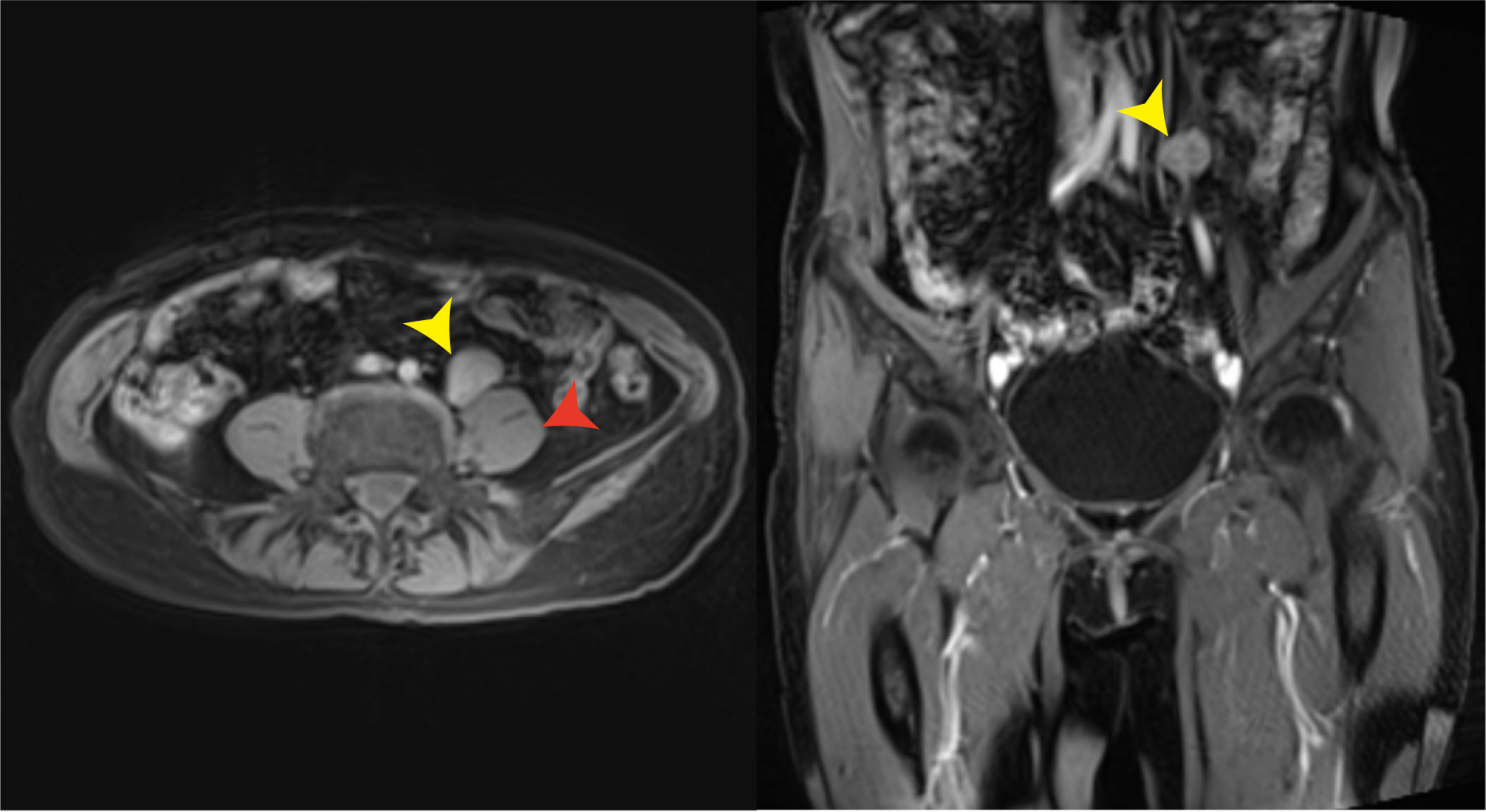
Pelvic MRI on 7 January 2022. A likely metastatic lymph node (yellow arrows) in the anterior area of the left psoas major (red arrow) and left of the common iliac artery was found 15 months after the administration of olaparib.

**Figure 4:**
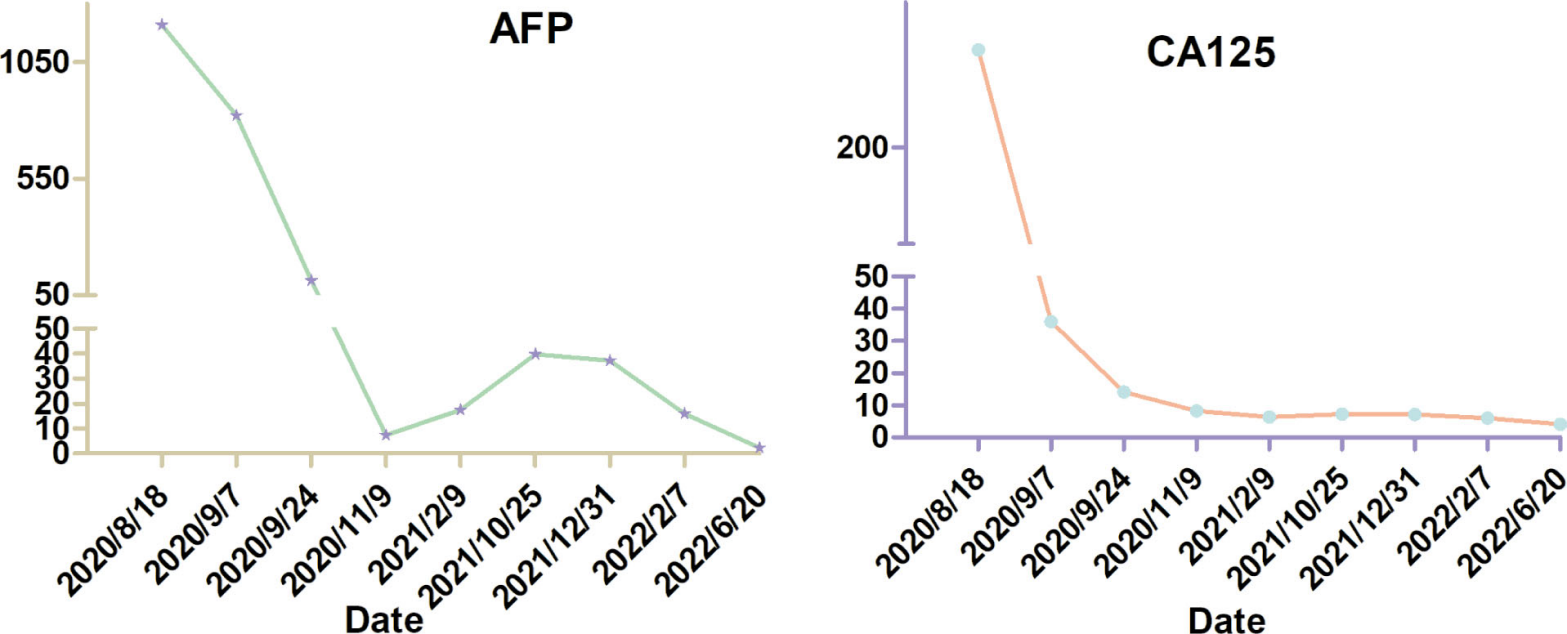
The level of alpha fetoprotein (AFP) and cancer antigen 125 (CA125).

It is unfortunate that the patient did not tolerate the side effects of olaparib. She now lives a relatively normal life with good quality. The levels of AFP and CA125 were dramatically reduced after the cytoreductive surgery. On 9 November 2020, 1.5 months after the administration of olaparib, the levels of AFP and CA125 decreased to normal. On 17 November 2020, enhanced CT scan of her upper abdomen revealed that the lesion above the pancreas was 1.4 cm × 1.5 cm × 2.0 cm, becoming smaller than the lesion size on the CT scan on 19 August 2020. However, a nodule with a diameter of 0.6 cm was seen in the omentum area, suggesting a possible metastasis. On 1 January 2022, the lesion above the pancreas shrunk to 1.0 cm × 1.4 cm and the nodule in the omentum area was not seen anymore. On 12 February 2021, the pelvic MRI scan showed no visible lesions after olaparib administration for more than 4 months. On 7 January 2022, 15 months after the administration of olaparib, the latest pelvic MRI scan discovered a nodule (2.5 cm in diameter) with the probability of a metastatic lymph node in the anterior area of the left psoas major and left of the common iliac artery (**Figure 3**). The levels of AFP and CA125 were also slightly elevated (15.90 U/ml and 6.07 ng/ml, respectively, on 7 February 2022) (**Figure 4**).

## Discussion

STOs account for 10%–25% of all ovarian malignancies (1). Owing to their heterogeneity and deficiency of relevant research, the available data concerning STOs are rather limited, and there are no uniform guidelines in the treatment of STOs. The usual primary tumors causing ovarian metastases are breast, colorectal, endometrial, stomach, and appendix cancer (1). The incidence rate of ovarian metastases from liver cancer is rather low. Our case is a sufficient supplement to the data of how to manage ovarian metastases.

In the case of our patient, we prepared genetic test with the patient’s consent after the operation, and the results showed that the patient carried mutations of BRAC2, RAD50, and PALB2. A recent study identified that approximately 25.8% (92/357) of patients with primary liver cancer had at least one DNA-damage response gene mutation, 4.8% (17/357) of whom carried *BRCA1/2* mutations (7). In accordance, olaparib showed promising clinical benefits for germline *BRCA1/2*-mutated intrahepatic cholangiocarcinoma (7). Recently, Johnston et al. (8) found that olaparib suppressed tumor growth of hepatoblastoma in patient-derived xenograft models. Zhao et al. (9) recently reported a patient with liver cancer carrying BRCA2 germline mutation who achieved stable disease and improvement in hepatalgia for 3 months after the combination treatment of olaparib and nivolumab. In 2017, the US Food and Drug Administration (FDA) approved olaparib as monotherapy in the maintenance therapy for patients with relapsed high-grade serous epithelial ovarian, fallopian tube, or primary peritoneal cancer, who were having a complete or partial response following platinum-based chemotherapy. Based on the above literature references and the specific conditions of the patient, we selected olaparib as experimental treatment and achieved good results. The present case has survived for nearly 2 years, and the lesion above the head of the pancreas was reduced. It is unfortunate that the patient did not tolerate the side effects of olaparib; otherwise, the patient would not have the sign of recurrence until now. The patient has been planned for close follow-up to further evaluate the extent of benefit from our medical plan.

There is no complete research on the way and the specific mechanism of how liver cancer cells migrate to the ovary. It is well known that the liver plays a vital role in the metabolism of carbohydrates, lipids, and proteins. Metastasis and metabolism reprogramming are two major hallmarks of cancer. Recent studies have shown that metabolic abnormalities in the tumor itself and its microenvironment are closely related to tumor metastasis. The progression of HCC is associated with inflammation and complex metabolic reprogramming. Defects in hepatic lipid metabolism induce abnormal gene expression and rewire many cellular pathways involved in oncogenesis and metastasis (10). Lin et al. (11) clarified that the positive feedback loop of Reactive Oxygen Species-Forkhead box C1-cysteine metabolism-ROS is important for promoting liver cancer proliferation and metastasis. As the major component of the tumor stroma, Cancer-associated Fibroblasts play critical roles in tumor initiation and metastasis by producing Extracellular Matrix-degrading enzymes and secreting growth factors and cytokines. Enhanced aerobic glycolysis has been demonstrated in CAFs. These CAFs support tumor cell survival and favor cancer metastasis by providing their glycolytic end-product lactate to neighboring cancer cells (12). Next, we will conduct further research on this rare case to explore the specific pathways and related mechanisms on how HCC cells metastasize to the ovary from the perspective of tumor metabolic reprogramming. This may help us to look for novel therapeutic approaches for this kind of diseases as well as STOs.

## Conclusions

STOs that originated from liver cancer are extremely rare. There is no consensus of opinions on the treatment of ovarian metastases owing to their different origins and biological behaviors, and survival time is generally short due to limited management options. Our experience in the management of the patient with metastatic ovarian adenocarcinoma that originated from liver cancer is a complement to the management of STOs that originated from the liver and other sites.

In our perspective, cytoreductive surgery is firstly important for the management of STOs. The levels of AFP and CA125 were dramatically reduced after the surgery in our case. Cytoreductive surgery can remove the tumor burden to the utmost. Meanwhile, genetic testing is also of great assistance in the management of patients with STOs, as it can help to elucidate the molecular signature of the tumor. We suggest that all patients with ovarian cancer or liver cancer should undergo genetic testing to help manage the malignancy. It is a pity that the patient did not tolerate olaparib, so that she only took the targeted drug for 2.5 months following surgery. However, it is enough for us to say that olaparib has antitumor activity against *BRCA2*, *RAD50*, and *PALB2*-mutated ovarian metastases that originated from liver cancer, as the levels of AFP and CA125 were reduced to normal after the administration of olaparib together with the shrinking of the lesion above the head of the pancreas. Therefore, we postulate that olaparib may be a good choice for patients with STOs or liver cancer harboring *BRCA2*, RAD50, or PALB2 mutation. It is worth noting that the patient has been taking traditional Chinese medicine to recuperate since the surgery of the primary liver cancer. Further clinical research is needed to explore the effectiveness of olaparib or other PARPi in the management of STOs or liver cancer and identify the molecular subgroups that are sensitive to PARPi.

## Data availability statement

The raw data supporting the conclusions of this article will be made available by the authors, without undue reservation.

## Ethics statement

Ethical review and approval was not required for the study on human participants in accordance with the local legislation and institutional requirements. The patients/participants provided their written informed consent to participate in this study. Written informed consent was obtained from the individual(s) for the publication of any potentially identifiable images or data included in this article.

## Author contributions

ZC and WHY contributed to the conception and design of the study. CL and WLY participated in collecting data and writing the first draft of the manuscript. ZY and WZ performed the statistical analysis and participated in the figure processing. All authors contributed to the article and approved the submitted version.
